# A challenging upper digestive tract continuity restoration after recurrent esophago-colonic anastomosis complications

**DOI:** 10.1186/s13019-022-02085-1

**Published:** 2022-12-17

**Authors:** Francesco Ferrante, Massimiliano Bassi, Daniele Diso, Rita Ferreira Vaz Sousa, Alessandro Maria Paganini, Federico Venuta, Tiziano De Giacomo

**Affiliations:** 1 Department of Thoracic Surgery, “Sapienza” University of Rome, Policlinico Umberto I Rome, Viale del Policlinico, 155, 00161 Rome, Italy; 2 Department of Bariatric Surgery, “Sapienza” University of Rome, Policlinico Umberto I Rome, Rome, Italy

**Keywords:** Esophago-colonic anastomosis, Anastomosis failure, Esophageal surgery, Digestive tract continuity

## Abstract

**Background:**

Acute and chronic complications in esophago-colonic anastomosis have a significant impact in the postoperative course of patients with colonic transposition. Evidence about their management is poor and surgical treatment is mostly based on tailored approaches, so each new experience could be useful to improve knowledge about this peculiar condition. We report a unique case of an esophago-colonic resection and re-anastomosis without sternal approximation after recurrent anastomosis failure and strictures.

**Case presentation:**

A 69-year-old woman was referred to our hospital for worsening dysphagia. The patient had undergone esophago-gastrectomy with right colon interposition 12 years prior due to caustic ingestion. The esophago-colonic anastomosis was initially complicated by an enterocutaneous fistula, which was treated with anastomosis resection and left colon transposition. This was then further complicated by dehiscence and sternal infection treated with resection of the distal portion of the sternum and a new colo-jejunal anastomosis. Finally, a chronic anastomotic stricture occurred, refractory to endoscopic dilatation and prothesis positioning. We planned a new colonic-esophageal resection and re-anastomosis. The main technical challenges were addressing the adhesions resulting from previous surgery and mobilizing an adequate length of the intestinal tract to allow conduit continuity restoration. Blood supply was assessed through Indocyanine Green Fluorescence. To avoid compression of the digestive conduit sternal margins were not re-approximated, and the transposed tube was covered and protected using both pectoralis major muscles flap. We decided to avoid the use of any prosthetic material to reduce the risk of infection. The patient was able to resume oral food intake on the 12th day postoperatively after a barium swallowing test showed an adequate conduit caliber.

**Conclusion:**

Esophago-colonic anastomosis complications represent a life-threatening condition. Therefore, reports and sharing of knowledge are important to improve expertise in management of these conditions.

## Introduction

Esophago-colonic anastomosis complications embrace a variety of chronic and acute disorders including redundancy, stricture, fistula, anastomotic dehiscence, and others [1]. Acute and chronic disorders occur respectively in 23% and 36% of cases [2]. There is a limited experience in international literature on their operative treatment due to the rarity and complexity of cases. Surgical treatment is technically demanding, but often represents the only chance to restore the upper digestive tract. General guidelines are lacking, and surgical strategies are often based on reports tailored to each patients’ disease and condition.

We describe a unique case of retrosternal esophago-colonic resection and re-anastomosis for chronic refractory stricture after recurrent dehiscence in a patient who had undergone esophago-gastrectomy 12 years prior due to caustic injury.

## Case report

A 69-year-old woman was referred to our hospital because of worsening dysphagia and weight loss in the last year. Twelve years prior the patient had undergone esophago-gastrectomy for caustic ingestion, at another institution. The restoration of the alimentary tract was obtained initially with a retrosternal right colon interposition and esophago-colonic anastomosis since the severity of the caustic injuries did not allow the use of a gastric tube. The postoperative course was characterized by esophago-colonic anastomotic leakage and entero-cutaneous fistula treated with antibiotics and cervical abscess drainage. Thus, the right colonic tract was removed and replaced with left colon transposition through a median sternotomy two months after the first operation. Over the years, the patient presented severe stricture of the colo-jejunal anastomosis, complicated with mediastinal and sternal infection due to a new entero-cutaneous fistula. This was treated surgically with resection of the distal portion of the sternum and a new colo-jejunal anastomosis. Since then, the patient underwent repeated endoscopic dilatation for secondary esophago-colonic anastomotic stricture. A self-expandable endoprosthesis was positioned for worsening dysphagia, however it was removed after 10 days due to severe patient’s discomfort and pain.

**Fig. 1:**
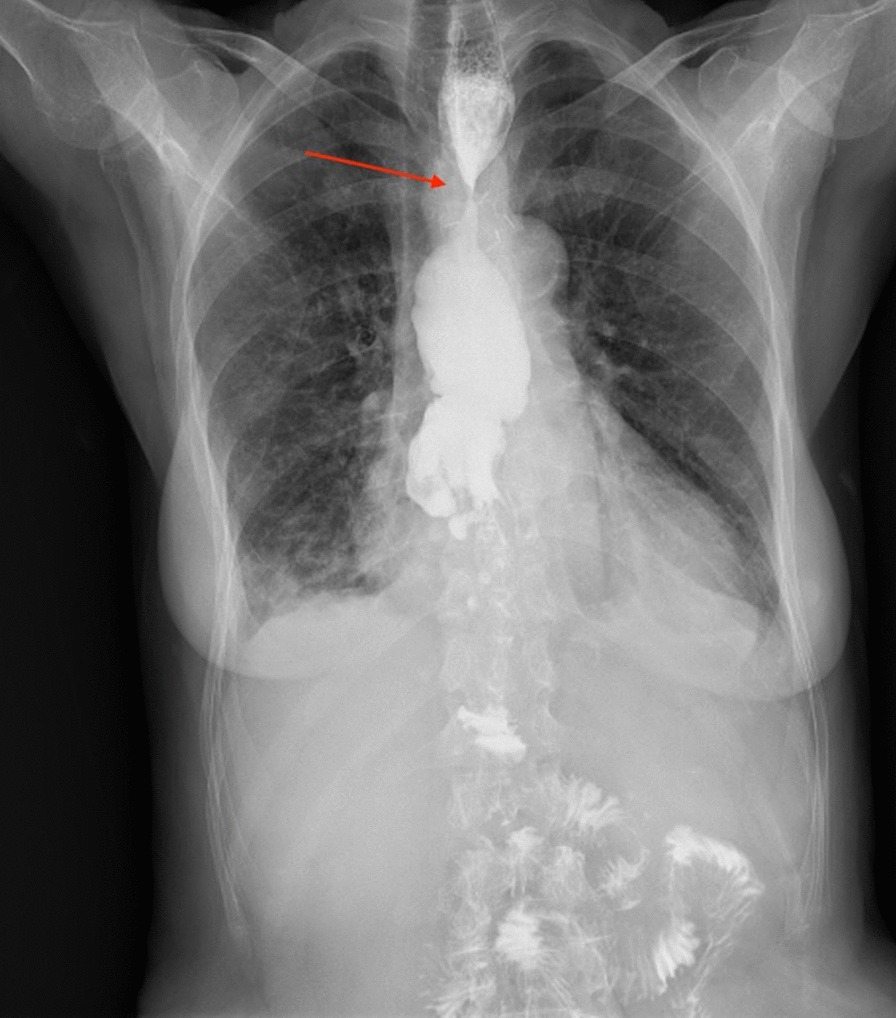
Preoperative barium swallow test shows severe esophago-colonic anastomosis stricture (red arrow) with proximal esophageal dilatation

Upon presentation at our hospital, the patient complained of severe dysphagia with almost no capability of oral intake. Body weight was 38 kg and body mass index 13.5. A barium swallow test revealed a severe stricture of nearly 90% of the esophageal lumen at the level of the esophago-colonic anastomosis. That completely precluded solid oral intake and severely compromised liquid intake, which resulted in frequent episodes of inhalation (Fig. 1). Endoscopy showed a severe and rigid stenosis with about 3–4 mm of residual caliber Considering the failure of endoscopic treatments, surgical esophago-colonic resection and re-anastomosis was planned to restore the upper digestive transit.

**Fig. 2:**
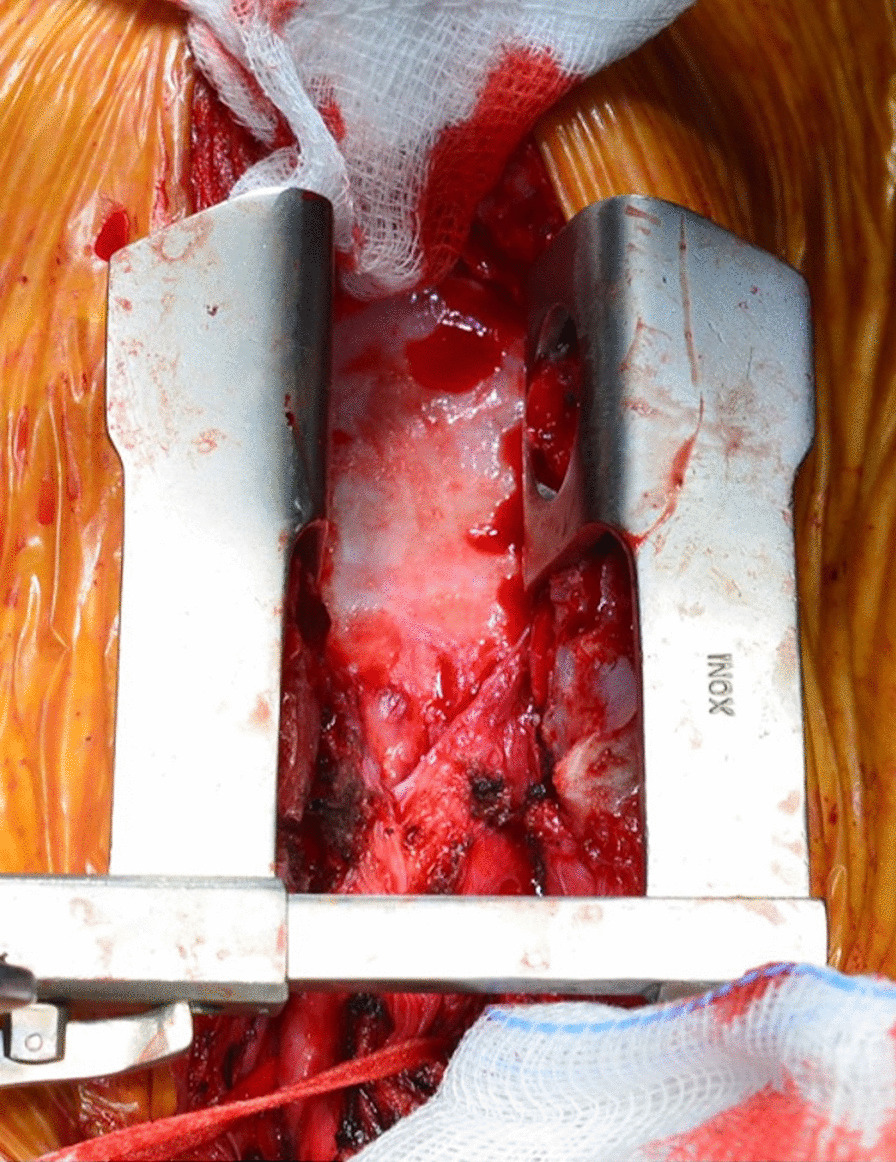
Intraoperative view. Fibrocalcific tissue takes up the whole anterior mediastinum after sternal opening

**Fig. 3:**
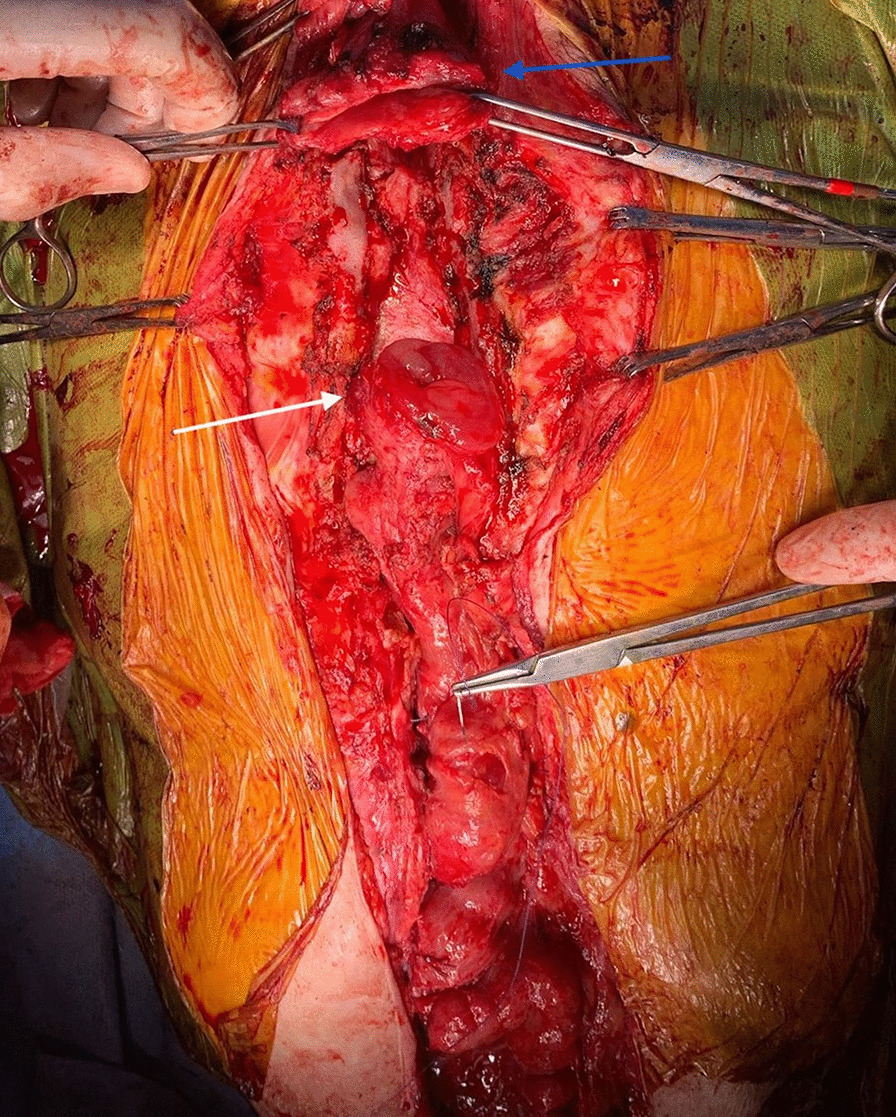
After intestinal loop mobilization, approximation of esophago-colonic ends is quite complete. Blue arrow shows esophageal stump, white arrow shows colonic stump

Re-median sternotomy was cautiously performed. The anastomotic tract appeared completely encased by fibro-calcific tissue probably due to chronic inflammation and previous surgery (Fig. 2). Since it was impossible to separate and free the stenotic tract from the mediastinal fibrocalcific tissue, the uninvolved portion of the transposed left colon was released from mediastinal adherences and isolated. Left colon and cervical esophagus were transected adjacent to the stenosis and after that a residual gap of 15 cm was present between the colonic and the esophageal stump. A cervical incision was performed in order to adequately prepare and release the proximal esophagus and a median laparotomy was performed to achieve adequate colon-jejunal mobilization. Extensive peritoneal adhesions were cautiously divided and mesenteric radial incisions were performed to allow transposition of the colon-jejunal tract as cranially as possible (Fig. 3). We took care to avoid excessive de-vascularization, and indocyanine green (ICG) fluorescence was used to assess the proper vascular supply of the cervical esophagus and the transposed intestinal tract. Finally, the esophageal and proximal colonic stumps were re-approximated without tension and the anastomosis was performed using a 3/0 non-absorbable suture. The sternal margins were not re-approximated to avoid compression of the digestive conduit between the extensive fibrocalcific tissue in the upper anterior mediastinum and the sternum. We decided to avoid the use of any prosthetic material for protecting the digestive conduit to reduce the risk of infection, but the anastomosis and the transposed tube were covered and protected using an adequately mobilized flap of both pectoralis major muscles.

**Fig. 4:**
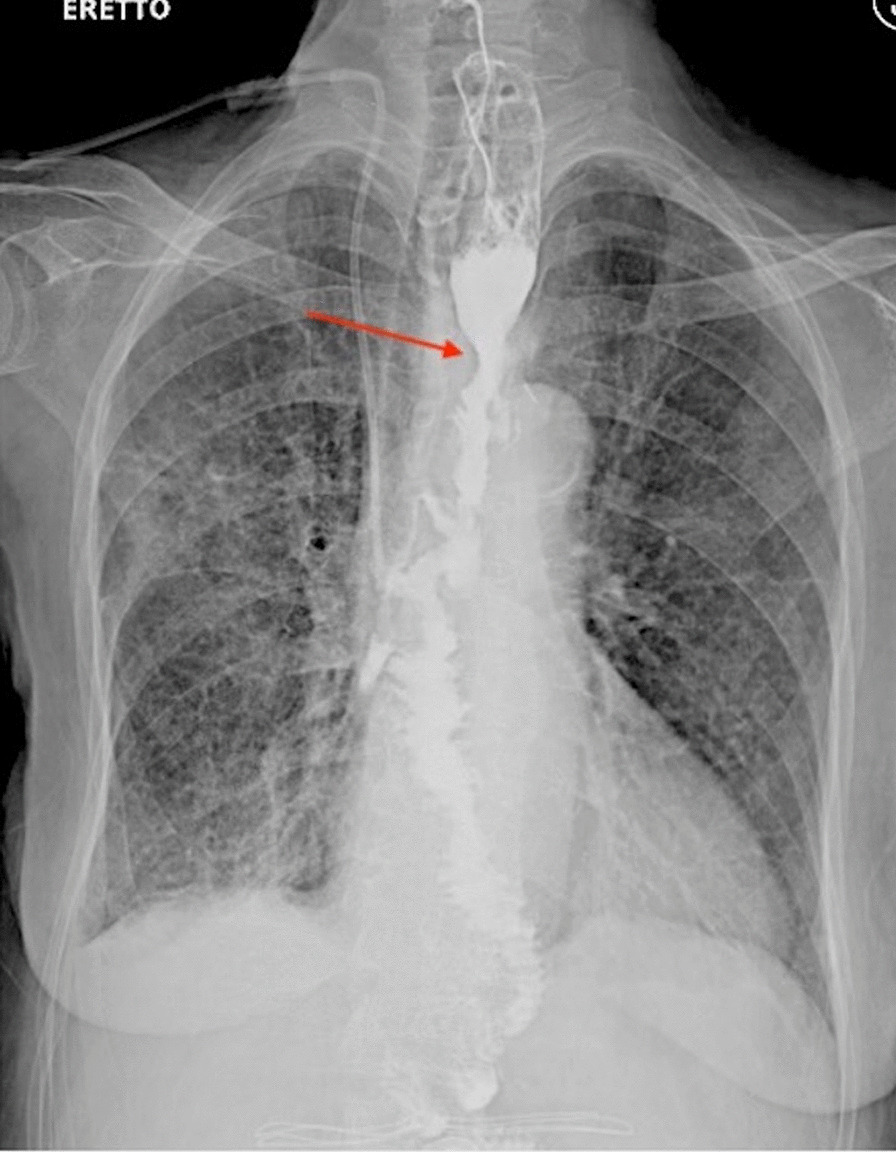
Postoperative barium swallow test points out a significant improvement in conduit caliber. Red arrow shows where re-anastomosis was performed

The postoperative course was characterized by Klebsiella pneumoniae pulmonary infection successfully treated with antibiotics. No anastomotic complication or chest wall instability were observed. The postoperative barium swallowing test performed on day 10 revealed a proper caliber of the upper digestive tract and absence of strictures and leaks (Fig. 4). Therefore, oral intake was started on postoperative day 12 and enteral nutrition was progressively reduced. The patient was discharged on postoperative day 36 and the jejunostomy was removed 20 days later. Six months after surgery nutrition status was significantly improved and no discomfort in oral intake was observed.

## Discussion

Retrosternal colonic transposition is considered a feasible and effective option in case of esophagectomy and gastrectomy for caustic injury [3]. Re-operation for complications of the esophago-colonic anastomosis is a demanding surgery, but often represents the last resource to restore the upper digestive tract [4]. Experience in international literature is lacking and limited to case reports or small case series mostly based on tailored approaches [1, 5–8].

Because of its exceptional nature, we propose some general tips that could be useful for further similar experiences:

*Caution in re-sternotomy* In case of re-sternotomy, fibrosis and tenacious mediastinal adhesions are expected. In order to avoid conduit damage, sternal division must be performed while carefully preserving underlying tissues.

*Tension-free anastomosis* Intestinal loops are often encased in fibrous tissue due to previous surgery and their mobilization could represent a surgical challenge. Radial cuts on the mesentery are useful to reduce tension between loops and their vascular arch to accomplish a tension free anastomosis. It is advisable to use vascular structure as an anatomic guide to avoid unintentional damage.

*ICG fluorescence for vascularization assessment* An adequate blood supply is crucial for the anastomosis outcome. ICG fluorescence could represent an effective way to assess vascularization of both colonic and esophageal ends.

*Avoid conduit compression* Due to previous sternotomy and fibrotic mediastinal adhesions, the risk of retrosternal conduit compression is high [9]; in this case we did not re-approximate sternal margins considering the stability and rigidity of the anterior chest wall due to the fibrocalcific tissue in the anterior mediastinum. In fact, in this case sternal closure would have caused significant compression on the conduit because of the extensive fibrosis and calcification of the mediastinum immediately under it. However, intestinal conduit requires protection. In this case, the high risk of anastomotic complications and infection bring us to avoid the use of prosthetic material. Pedicled muscle flap coverage with native pectoralis major muscles was used.

## Conclusion

Esophago-colonic anastomosis complications represent a life-threatening condition and a challenge for surgeons. Due to the exceptionality of cases, highly personalized treatment is required. Reports of single cases and sharing of experiences could be helpful in the management of future similar circumstances.

## Data Availability

Data sharing not applicable to this article as no dataset was generated for the study.

## Abbreviation

ICG: Indocianine green
